# Nasal Carriage of Methicillin-Resistant *Staphylococcus aureus* among Healthcare Workers in a Tertiary Care Hospital, Kathmandu, Nepal

**DOI:** 10.1155/2021/8825746

**Published:** 2021-08-10

**Authors:** Nisha Giri, Sujina Maharjan, Tika Bahadur Thapa, Sushant Pokhrel, Govardhan Joshi, Ojaswee Shrestha, Nabina Shrestha, Basista Prasad Rijal

**Affiliations:** ^1^Department of Laboratory Medicine, Manmohan Memorial Institute of Health Sciences, Soalteemode, Kathmandu, Nepal; ^2^Department of Clinical Biochemistry, Dhulikhel Hospital, Kavrepalanchowk, Dhulikhel, Nepal; ^3^Department of Genetics Laboratory, National Academy of Medical Sciences, Bir Hospital, Kathmandu, Nepal; ^4^Department of Clinical Microbiology, Manmohan Memorial Medical College and Teaching Hospital, Swoyambhu, Kathmandu, Nepal

## Abstract

**Introduction:**

Methicillin-resistant *Staphylococcus aureus* (MRSA) is one of the most common causes of nosocomial infections. One of the potential risk factors for nosocomial staphylococcal infections is colonization of the anterior nares of healthcare workers (HCWs). Our study aimed to determine the rate of nasal carriage MRSA among HCWs at Manmohan Memorial Medical College and Teaching Hospital, Kathmandu.

**Methods:**

Two hundred and thirty-two nasal swabs were collected from HCWs of Manmohan Memorial Medical College and Teaching Hospital, Kathmandu, Nepal, within six months (February 2018–July 2018). Nasal swabs were cultured, and *S. aureus* isolates were subjected to the antimicrobial susceptibility test by the modified Kirby–Bauer disc diffusion method. MRSA and iMLSB (inducible macrolide lincosamide streptogramin B) resistance was screened using the cefoxitin disc (30 *μ*g) and D-test (clindamycin and erythromycin sensitivity pattern), respectively, following CLSI (Clinical and Laboratory Standard Institute) guidelines. Risk factors for MRSA colonization were determined using the chi-square test considering the *p* value ˂0.05 as significant.

**Results:**

A total of 34/232 (14.7%) *S. aureus* were isolated, out of which 12 (35.3%) were MRSA. The overall rate of nasal carriage MRSA among HCWs was 5.2% (12/232). Colonization of MRSA was higher in males (8.7%) than in females (4.3%). MRSA colonization was found to be at peak among the doctors (11.4%). HCWs of the postoperative ward were colonized highest (18.2%). All MRSA isolates were sensitive to linezolid and tetracycline. iMLSB resistance was shown by 7(20.6%) of the isolates. MRSA strains showed higher iMLSB resistance accounting for 33.3% (4/12) in comparison to methicillin-susceptible strains with 13.6% (3/22). Smoking was found to be significantly associated with MRSA colonization (*p*=0.004).

**Conclusion:**

Rate of nasal carriage MRSA is high among HCWs and hence needs special attention to prevent HCW-associated infections that may result due to nasal colonization.

## 1. Introduction

*Staphylococcus aureus* is a major human pathogen which has potential to cause ample of clinical infections ranging from bacteraemia and infective endocarditis to osteoarticular, skin and soft tissue, pleuropulmonary, and device-related infections [1]. With a few exceptions, the incidence of hospital-acquired infection caused by methicillin-resistant *Staphylococcus aureus* (MRSA) is increasing worldwide, resulting in longer hospital stay, prolonged antibiotic administration, and higher costs [2].

Penicillin was introduced in the 1940s to treat staphylococcal infections; however, in 1945, strains of *S. aureus* developed resistance to it. Later, methicillin was introduced in 1959, but in 1961, *S. aureus* isolates acquired resistance to methicillin as well [3]. MRSA strains exhibit blanket resistance to virtually all *β*-lactams, often associated with resistance to other classes of antibiotics [4]. The *mecA* gene, present on the staphylococcal cassette chromosome (SCC), codes for a low antibiotic affinity PBP (penicillin-binding protein) known as PBP2a, responsible for the resistance among MRSA strains [5].

A few options are available for the treatment of MRSA-associated infections, such as macrolides, lincosamides, and streptogramin B (MLSB) with clindamycin being a good alternative, particularly for skin and soft tissue infections, and also works as an alternative in penicillin-allergic patients [6]. However, MLSB resistance is one of the most common resistance mechanisms detected in Gram-positive organism [7]. MLSB resistance can be either constitutive (cMLSB) or inducible (iMLSB) [8]. Failure in detection of iMLSB resistance may be a clinical failure of clindamycin treatment [9]. Since the incidence of inducible clindamycin resistance is high, accurate identification of inducible clindamycin resistance is important to prevent therapeutic failure in infections caused by these strains [10].

It has been reported that HCWs have been the source of MRSA outbreaks in several cases [11]. *S. aureus* nasal carrier, a nurse, caused outbreaks in two newborn nurseries at different hospitals in association with upper respiratory tract infections in 1986. Phage typing revealed that the nurse's strain of *S. aureus* and the outbreak strains were identical [12]. Previous studies suggest possibilities that HCWs play a substantial role in MRSA transmission, highlighting the importance of rapid and accurate identification of MRSA carrier HCWs [13]. *S. aureus* nasal carriage among the general adult population shows global variation [14]. The knowledge of frequency of nasal carriage *S. aureus* and MRSA among HCWs along with their current antimicrobial profile becomes necessary in the selection of appropriate treatment options for these carriers. Several studies have shown that the elimination of carriage in the anterior nares reduces the incidence of staphylococcal infections [15].

Hence, this study was carried out to determine the rate of nasal carriage *S. aureus* and MRSA along with their antimicrobial profile among HCWs. Also, this study aimed to determine the associated risk factors for nasal carriage MRSA.

## 2. Materials and Methods

### 2.1. Study Design

A hospital-based cross-sectional study was performed in the Department of Microbiology of Manmohan Memorial Medical College and Teaching Hospital, Kathmandu, Nepal, within six months (February 2018–July 2018) among 238 HCWs with informed written consent. However, six HCWs did not provide consent, and hence, only 232 HCWs were included in the study. All the participants in the study were interviewed with a standard questionnaire for the clinical history and demographic data.

### 2.2. Inclusion Criteria

All the hospital staff who provided patient care directly were involved in the study.

### 2.3. Exclusion Criteria

HCWs presented with wound and upper respiratory tract infection in the last three months were excluded from the study.

### 2.4. Nasal Swab Collection

Nasal swabs were collected using sterile cotton swab moistened with normal saline. The swab was introduced 1-2 cm in the nasal cavity and rotated 3 times both clockwise and anticlockwise. For each specimen, both nostrils were sampled using the same swab and immediately transported to the laboratory on peptone water. The swabs, after 4 hours of incubation at 37°C on peptone water, were inoculated on mannitol salt agar.

### 2.5. Identification of *S. aureus*

*S. aureus* was isolated using mannitol salt agar. The isolates were identified by examination of colony characteristics, Gram staining, oxidase test, catalase test, slide coagulase test, tube coagulase test, and deoxyribonuclease test.

### 2.6. Detection of MRSA

All isolated *S. aureus* were tested with 30 *μ*g cefoxitin on Muller Hinton Agar (MHA) for MRSA screening. The zone size was interpreted according to CLSI guidelines. An inhibition zone diameter of ≤21 mm was reported as MRSA and ≥22 mm was reported as methicillin-sensitive *Staphylococcus aureus* (MSSA).

### 2.7. Antibiotic Susceptibility Testing

Antibiotic susceptibility testing was performed by the modified Kirby–Bauer disc diffusion method on MHA using standard methods as recommended by CLSI guidelines. The antibiotics tested were amikacin (30 *μ*g), amoxicillin (10 *μ*g), cefepime (30 *μ*g), cefotaxime (30 *μ*g), cefoxitin (30 *μ*g), chloramphenicol (30 *μ*g), ciprofloxacin (5 *μ*g), clindamycin (2 *μ*g), cloxacillin (10 *μ*g), cotrimoxazole (25 *μ*g), erythromycin (15 *μ*g), gentamicin (10 *μ*g), linezolid (30 *μ*g), ofloxacin (5 *μ*g), teicoplanin (30 *μ*g), and tetracycline (30 *μ*g). The result was interpreted as per the guidelines of CLSI zone size interpretative chart in terms of “sensitive,” “resistant,” and “intermediate sensitive.”

### 2.8. Detection of iMLSB Resistance

Erythromycin (E) and clindamycin (CD) sensitivity patterns were reported following CLSI guidelines. Isolates were tested for inducible resistance using the D-test. For the detection of iMLSB resistance, 0.5 McFarland equivalent suspension of organism was inoculated into MHA plate as described in the CLSI recommendation. CD (2 *μ*g) and E (15 *μ*g) discs were placed 15 mm apart from the center on the MHA. Plates were analyzed after 18 hours of incubation at 37°C. If the E zone was ≤13 mm and the CD zone was ≥21 mm and both had a circular shape, the organism was negative for inducible resistance (D-test negative). If the E zone was ≤13 mm and the CD zone was ≥21 mm with a D-shaped zone around the CD, the organism was positive for inducible resistance (D-test positive).

### 2.9. Data Analysis

Each sample was encoded with an identification number. Similarly, findings were recorded manually and entered into database. The analysis was carried out by SPSS version 20 (IBM Corporation, Armonk, NY, USA). Risk factors for MRSA colonization were determined using the chi-square test considering the *p* value ˂0.05 as significant.

## 3. Results

**Figure 1:**
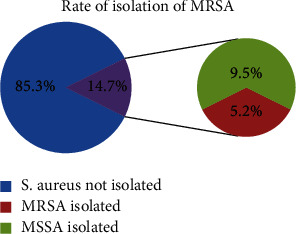
Rate of isolation of MRSA.

A total of 232 nasal swabs were collected from HCWs, of which 34 (14.7%) were found to be carriers of *S. aureus*, and among them, 12/34 (35.3%) HCWs were found to be MRSA carriers. Overall distribution of MRSA was found to be 5.2% (12/232) (Figure 1).

### 3.1. Demographic Characteristics of the Study Population

The age of study participants ranged from 16 to 65 years, with a mean age of 28.71 ± 7.8 years. The age group of 25–35 years (47.8%) participated in the study was in higher frequency followed by <25 years (34.1%), 35–45 years (13.4%), and least with the age group of ≥45 years (4.7%). Females were predominantly in higher fractions (80.2%) in the present study (Table 1).

### 3.2. Distribution of Bacterial Isolates

Distribution of MSSA was the highest in HCWs of the age group 25–35 years with 11.7% (13/111) and MRSA in the age group ≥45 years with 9.1% (1/11). MSSA and MRSA were found to be 19.6% (9/46) and 8.7% (4/46), respectively, in males being higher in comparison to females (Table 1).

Similarly, the prevalence of MSSA was found to be the highest among laboratory staffs with 25% (5/20), while MRSA was found to be the highest among doctors with 11.4% (5/44) (Table 2).

Distribution of MSSA was found to be highest in laboratory staffs with 19.2% (5/26), while MRSA was found to be the highest in postoperative ward staffs with 18.2% (2/11) (Table 3).

### 3.3. Antibiotic Susceptibility Pattern of *S. aureus*

Among 34 isolated *S. aureus*, 12 were methicillin-resistant, whereas 22 were methicillin-sensitive. Beside penicillins and cephalosporins, MRSA strains were lowest sensitive to ofloxacin and ciprofloxacin (25% each), followed by erythromycin (33.3%) and gentamicin (50%). However, all MRSA strains were sensitive to linezolid and tetracycline, highest sensitivity followed by teicoplanin and chloramphenicol (91.7% each), amikacin and clindamycin (83.3% each), and cotrimoxazole (75%).

**Figure 2:**
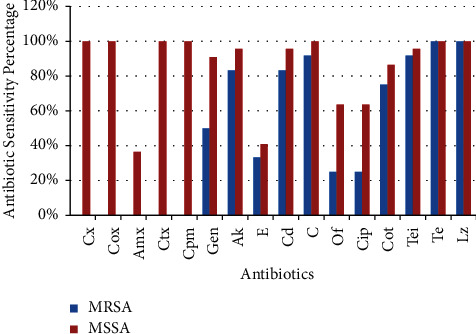
Comparison of antibiotic susceptibility pattern between MRSA and MSSA. Cx, cefoxitin; Cox, cloxacillin; Amx, amoxicillin; Ctx, cefotaxime; Cpm, cefepime; Gen, gentamicin; Ak, amikacin; E, erythromycin; Cd, clindamycin; C, chloramphenicol; Of, ofloxacin; Cip, ciprofloxacin; Cot, cotrimoxazole; Tei, teicoplanin; Te, tetracycline; Lz, linezolid.

Among MSSA, the lowest sensitivity was found for amoxicillin (36.4%), followed by erythromycin (40.9%) and ofloxacin and ciprofloxacin each being equally sensitive (63.6%). All isolated MSSA strains were found sensitive to cloxacillin, cefotaxime, cefepime, and chloramphenicol in addition to linezolid and tetracycline. MSSA also showed higher sensitivity to amikacin, clindamycin and teicoplanin (95.5% each), and gentamicin (90.9%) (Figure 2).

### 3.4. Erythromycin and Clindamycin Susceptibility Pattern of *S. aureus*

Among 34 isolates, 12 (35.3%) were sensitive to both erythromycin and clindamycin and 2 (5.9%) were resistant to both erythromycin and clindamycin. Similarly, 7 (20.6%) were resistant to erythromycin and sensitive to clindamycin with the D-test being positive, whereas 13 (38.2%) were sensitive to erythromycin and resistant to clindamycin but with the D-test negative. Our study showed higher iMLSB resistance in MRSA strains with 33.3% (4/12) in comparison to MSSA strains with 13.6% (3/22). A total of 7/34 (20.6%) of the isolates showed iMLSB resistance (Table 4).

### 3.5. Determining Risk Factors for MRSA Colonization

Smoking was found to be significantly associated with MRSA colonization (*p*=0.004). No other factors were significantly associated with MRSA colonization (Table 5).

## 4. Discussion

*S. aureus* is one of the most common multidrug resistant Gram-positive bacteria and perhaps the pathogen of greatest concern because of its intrinsic virulence, its ability to cause a diverse array of life-threatening infections, and its capacity to adapt to different environmental conditions [16]. In our study, the rate of nasal carriage *S. aureus* was found to be 14.7%, which is similar to the findings of Khanal et al. (15.7%) [17] and Khatri et al. (18.3%) in Nepal [18] and Legese et al. (12.0%) in Ethiopia [19]. In contrast, our study showed a lower prevalence of nasal carriage *S. aureus* in comparison with the study performed by Vaidya et al. in India (22%) [20], Chen et al. in China (21.6%) [21], Boncompain et al. in Argentina (30%) [22], Moshtagheian et al. in Iran (23.4%) [23], and several other studies [24–26].

In our study, the overall rate of nasal carriage MRSA was found to be 5.2%. Similar results were found in study performed by Vaidya et al. in India (6%) [20], Boncompain et al. in Argentina (6.3%) [22], and other studies [17–19]. However, the prevalence of nasal carriage MRSA in HCWs was found comparatively to be lower in our study in comparison to a study performed in the Gaza strip by Aila et al. (25.5%) [26]. Whereas, the prevalence of MRSA was found to be slightly higher in our study in comparison to findings of Shrestha et al. in Nepal (2.3%) [27], Chen et al. in China (1.0%) [21] and Peters et al. in Germany (1.6%) [28]. Such differences in the rate of nasal carriage *S. aureus* and MRSA among HCWs may be attributed to different factors such as sampling techniques, culture methods, identification of *S. aureus* and MRSA basis, study population, study criteria, and hospital environment.

Our study also reported that colonization of MRSA was high in males (8.7%) than in females (4.3%) (*p* > 0.05). Similarly, Boncompain et al. reported higher MRSA colonization in males (7.2%) in comparison to females (5.8%) in Argentina [22], while Khatri et al. reported that colonization was higher in females (8.3%) in comparison to males (5.1%) in Nepal [18].

Profession wise, our study showed the highest MRSA colonization of 11.4% among doctors. Similarly, Vaidya et al. in India reported that doctors were colonized the highest with MRSA [20]. While, Khatri et al. in Nepal reported that lab personnel were the highest nasal carriers of MRSA (10.5%) [18]. However, the study performed by Haftom et al. in Ethiopia reported MRSA carriage the highest among nurses accounting for 7.8% [19]. Higher colonization of MRSA among doctors may be due to frequent patient contact. The high rate of nasal carriage MRSA among HCWs indicates high chances of transmission of the pathogen to the patients during patient care [18], which ultimately leads to longer hospital stay, prolonged antibiotic administration, and higher costs.

Ward wise, our study showed that HCWs of the postoperative ward were colonized the highest (18.2%). This could be due to the traumatic and postoperative immunological suppression of the patients [29]. As most isolates belonged to HCWs from the postoperative ward, the vulnerability of surgical site wound infection with MRSA among patients, following transmission from carrier HCWs, cannot be ignored. Similarly, Khatri et al. in Nepal reported that the percentages of nasal carriage MRSA (14.3%) were the highest among HCWs from postoperative department [18]. However, Vaidya et al. in India reported that HCWs of the emergency ward were found to be colonized the highest (15.3%) [20]. In contrast, a study performed by Haftom et al. in Ethiopia reported that HCWs of the surgical ward were the highest colonized with MRSA (17.1%) [19].

In our study, all isolated *S. aureus* (both MRSA and MSSA) were sensitive to linezolid and tetracycline. However, in a study performed by Karimi et al., only 58.4% of *S. aureus* was sensitive to tetracycline [30]. MRSA strains also showed higher sensitivity to teicoplanin (91.7%), chloramphenicol (91.7%), amikacin (83.3%), and clindamycin (83.3%) in our study. High sensitivity of those antibiotics towards MRSA indicates that these antibiotics might be an option for empirical therapy of MRSA infections in our hospital. Similarly, all isolated MSSA were sensitive to chloramphenicol besides cloxacillin, cefoxitin, cefotaxime, and cefepime, followed by teicoplanin (95.5%), amikacin (95.5%), clindamycin (95.5%), gentamicin (90.9%), and cotrimoxazole (86.4%). Similarly, the abovementioned antibiotics might be implicated for the empirical therapy of MSSA infections in our hospital.

Among 34 isolates, 7 (20.6%) were resistant to erythromycin and sensitive to clindamycin with the D-test being positive. D-test positive was higher (33.3%) in MRSA in comparison to MSSA (13.6%). Our study showed lower iMLSB resistance (33.3%) among MRSA strains in comparison to a study performed by Khanal et al. in Nepal (66.7%) [17]. The differences in findings may be due to various factors such as different study populations, different study periods, and different settings and variations in methodology. Whereas, in a study performed by Adhikari et al. in Nepal, in clinical samples, iMLSB resistance and cMLSB resistance were found to be 11.48% and 29.25%, respectively. Comparatively, iMLSB resistance was found to be higher and cMLSB lower in our study [31]. The differences in findings may be due to differences in samples (nasal swabs vs. clinical samples).

Our study also aimed to determine the risk factors for MRSA colonization. The colonization of MRSA was significantly high among smokers (*p*=0.004). Cigarette smoking increases mucus production, impairs epithelial elastic properties, decreases IgA production, and affects phagocyte activities which facilitate bacterial colonization and exacerbate inflammatory responses leading to epithelial damage, further impairing host immunity and promoting bacterial colonization of the respiratory tract [32]. Coinciding with our study, smoking was found to be one of the risk factors for colonization among healthy adults [33].

In our study, other factors were not found to be significantly associated with MRSA colonization. However, years of service and level of education were found to be significantly associated with MRSA colonization in a study performed by Maroof et al. in India [34].

Regular surveillance and decolonization of MRSA-positive HCWs can help as an effective measure to control MRSA infection. Similarly, an awareness campaign in the HCWs may be reinforced to take adequate care and precautions regarding the universal techniques of hand washing and hygiene thereby minimizing risks of transmitting hospital-acquired MRSA. Furthermore, the association between carriage and infections can be established by identifying strains with the same genotype that helps to add further evidence on the relationship between nasal carriage MRSA among HCWs and incidence of staphylococcal infections among patients.

This study is based only on the phenotypic methods. It reveals only about the carriage status of *S. aureus* (carriage status of other organisms such as coagulase-negative *S. aureus* was not studied). Also, this study only determines the carriage status but does not suggest about the ways for its decolonization.

## 5. Conclusion

Our study revealed that the nasal carriage *S. aureus* and MRSA is high among HCWs, especially among the doctors and the HCWs of the postoperative ward. iMLSB resistance is higher in MRSA than in MSSA strains. The high rate of nasal carriage MRSA among HCWs indicates the need for standard infection control precautions to be employed in the professional practice to minimize the carriage as well as the transmission rate. Our study also suggested that smoking is significantly associated with MRSA colonization.

## Figures and Tables

**Table 1:** Distribution of *S. aureus* by age and sex.

Variable	Nasal carriage	Total sample, *n* (%)	*S. aureus*		
			Carriers		Noncarriers, *n* (%)
			MSSA, *n* (%)	MRSA, *n* (%)	
Age (years)	<25	79 (34.1)	5 (6.3)	4 (5.1)	70 (88.6)
	25–35	111 (47.8)	13 (11.7)	6 (5.4)	92 (82.8)
	35–45	31 (13.4)	3 (9.7)	1 (3.2)	27 (87.1)
	≥45	11 (4.7)	1 (9.1)	1 (9.1)	9 (81.8)
	Total	232 (100)	22 (9.5)	12 (5.2)	198 (85.3)

Gender	Male	46 (19.8)	9 (19.6)	4 (8.7)	33 (71.7)
	Female	186 (80.2)	13 (6.9)	8 (4.3)	165 (88.7)
	Total	232 (100)	22 (9.5)	12 (5.2)	198 (85.3)

**Table 2:** Profession wise distribution of *S. aureus*.

Profession	Total sample, *n* (%)	*S. aureus*		
		Carriers		Noncarriers, *n* (%)
		MSSA, *n* (%)	MRSA, *n* (%)	
Doctor	44 (18.9)	7 (15.9)	5 (11.4)	32 (72.7)
Laboratory personnel	20 (8.6)	5 (25)	0 (0)	15 (75.0)
Nurse	106 (45.7)	4 (3.8)	5 (4.7)	97 (91.5)
Attender	29 (12.5)	4 (13.8)	1 (3.4)	24 (82.8)
Cleaner	20 (8.6)	1 (5)	1 (5)	18 (90.0)
ANM	2 (0.9)	0 (0)	0 (0)	2 (100.0)
HA	6 (2.6)	1 (16.7)	0 (0)	5 (83.3)
CMA	5 (2.2)	0 (0)	0 (0)	5 (100)
Total	232 (100)	22 (9.5)	12 (5.2)	198 (85.3)

ANM, auxiliary nurse midwifery; HA, health assistant; CMA, certified medical assistant.

**Table 3:** Ward wise distribution of *S. aureus*.

Ward	Total sample, *n* (%)	*S. aureus*		
		Carriers		Noncarriers, *n* (%)
		MSSA, *n* (%)	MRSA, *n* (%)	
Laboratory	26 (11.2)	5 (19.2)	1 (3.8)	20 (76.9)
Medical	24 (10.3)	1 (4.2)	1 (4.2)	22 (91.6)
Cabin	13 (5.6)	1 (7.7)	1 (7.7)	11 (84.6)
Gynaecology	14 (6.0)	1 (7.1)	1 (7.1)	12 (85.7)
ICU	22 (9.5)	0 (0)	1 (4.5)	21 (95.5)
OT	15 (6.5)	2 (13.8)	1 (6.7)	12 (80.0)
Postoperative	11 (4.7)	2 (18.2)	2 (18.2)	7 (63.6)
Emergency	32 (13.8)	2 (6.3)	1 (3.1)	29 (90.6)
Surgical	27 (11.6)	1 (3.7)	0 (0)	26 (96.3)
OPD	43 (18.5)	7 (16.3)	3 (6.9)	33 (76.7)
Laundry/CSSD	5 (2.2)	0 (0)	0 (0)	5 (100)
Total	232 (100)	22 (9.5)	12 (5.2)	198 (85.3)

ICU, intensive care unit; OT, operation theatre; OPD, outpatient department; CSSD, central sterile services department.

**Table 4:** Erythromycin and clindamycin susceptibility pattern of *S. aureus*.

Resistance pattern	*S. aureus*, *n* (%)	MRSA, *n* (%)	MSSA, *n* (%)
E-S, CD-S	12 (35.3)	3 (25)	9 (40.9)
E-R, CD-R	2 (5.9)	1 (8.4)	1 (4.5)
E-R, CD-S (D+)	7 (20.6)	4 (33.3)	3 (13.6)
E-R, CD-S (D−)	13 (38.2)	4 (33.3)	9 (40.9)
Total	34 (100)	12 (100)	22 (100)

E, erythromycin; CD, clindamycin; S, sensitive; R, resistance; (D+), D-test positive; (D−), D-test negative.

**Table 5:** Analysis of potential risk factors associated with MRSA colonization.

Variable	Categories	Total sample (*n* = 232)	MSSA (*n* = 22)	MRSA (*n* = 12)	*P* value
Age (years)	<25	79	5 (6.3%)	4 (5.1%)	0.845
	25–35	111	13 (11.7%)	6 (5.4%)	
	35–45	31	3 (9.7%)	1 (3.2%)	
	≥45	11	1 (9.1%)	1 (9.1%)	

Sex	Male	46	9 (19.6%)	4 (8.7%)	0.664
	Female	186	13 (6.9%)	8 (4.3%)	

Associated ward	Laboratory	26	5 (19.2%)	1 (3.8%)	0.885
	Medical	24	1 (4.2%)	1 (4.2%)	
	Cabin	13	1 (7.7%)	1 (7.7%)	
	Gynaecology	14	1 (7.1%)	1 (7.1%)	
	ICU	22	0	1 (4.5%)	
	OT	15	2 (13.8%)	1 (6.7%)	
	Postoperative	11	2 (18.2%)	2 (18.2%)	
	Emergency	32	2 (6.3%)	1 (3.1%)	
	Surgical	27	1 (3.7%)	0	
	OPD	43	7 (16.3%)	3 (6.9%)	
	Laundry/CSSD	5	0	0	

Occupation	Doctor	44	7 (15.9%)	5 (11.4%)	0.326
	Lab technicians	20	5 (25%)	0	
	Nurse	106	4 (3.8%)	5 (4.7%)	
	Attender	29	4 (13.8%)	1 (3.4%)	
	Cleaner	20	1 (5.1%)	1 (5.0%)	
	ANM	2	0	0	
	HA	6	1 (16.7%)	0	
	CMA	5	0	0	

Years of service (years)	<1	65	2 (4.5%)	4 (9.1%)	0.523
	1–5	113	8 (7.5%)	3 (2.8%)	
	6–10	31	7 (13.2%)	3 (5.7%)	
	11–15	13	3 (20%)	1 (6.7%)	
	>15	10	2 (14.3%)	1 (7.1%)	

History of recurrent URTI (3 months before)	Yes	63	6 (9.5%)	6 (9.5%)	0.185
	No	169	16 (9.5%)	6 (3.6%)	

Nasal medication	Yes	14	1 (7.14%)	0	0.453
	No	218	21 (9.6%)	12 (5.5%)	

Nasal abnormalities	Yes	11	1 (9.1%)	0	0.453
	No	221	21 (9.5%)	12 (5.4%)	

Patient contact	Yes	185	16 (8.6%)	11 (5.9%)	0.192
	No	47	6 (12.8%)	1 (2.1%)	

Infection control training	Yes	7	1 (14.3%)	1 (14.3%)	0.645
	No	225	21 (9.3%)	11 (4.9%)	

Level of education	Illiterate	15	1 (6.7%)	1 (6.7%)	0.744
	Undergraduate	141	12 (8.5%)	5 (3.5%)	
	Graduate	73	9 (12.3%)	6 (8.2%)	
	Postgraduate	3	0	0	

History of hospitalization for >24 h	Yes	80	6 (7.5%)	3 (3.8%)	0.886
	No	152	16 (10.5%)	9 (5.9%)	

Intake of antibiotics within 3 months	Yes	38	5 (13.2%)	1 (2.6%)	0.293
	No	194	17 (8.8%)	11 (5.7%)	

Smoking habits	Yes	11	0	4 (36.4%)	**0.004**
	No	221	22 (9.9%)	8 (3.6%)	

MSSA, methicillin-sensitive *Staphylococcus aureus*; MRSA, methicillin-resistant *Staphylococcus aureus*; OR, odds ratio; CI, confidence interval; ICU, intensive care unit; OT, operation theatre; ER, emergency; OPD, outpatient department; CSSD, central sterile services department; ANM, auxiliary nurse midwifery; HA, health assistant; CMA, certified medical assistant; URTI, upper respiratory tract infectionBold face indicates *p* value <0.05.

## Data Availability

The data generated to support the findings of this study are included within the article. The primary raw data are available to interested researchers from the corresponding author upon request.
